# A rare case of intramuscular hemangioma of splenius capitis: a case report

**DOI:** 10.1097/MS9.0000000000000394

**Published:** 2023-03-27

**Authors:** Suman Maharjan, Anil Hona, Sabin Karki

**Affiliations:** a Nepalese Army Institute of Health Sciences (NAIHS), College of Medicine, Sanobharyang; b Department of Surgery, Shree Birendra Hospital, Chhauni, Kathmandu, Nepal

**Keywords:** case report, intramuscular hemangioma, splenius capitis, wide excision

## Abstract

Intramuscular hemangiomas are benign lesion that rarely occurs within the skeletal muscle of the head and neck regions. These lesions present with nonspecific symptoms resulting in only a few instances of accurate preoperative diagnosis.

**Case Presentation:**

A 20-year-old male presented with swelling over the nape of the neck on the right side. On clinical examination, the solitary swelling was 4×4 cm on measurement, soft on consistency with regular margin, fluctuant, with no skin changes over the swelling, nontender, no restriction in range of motion of the neck, and no pulsation felt.

**Clinical Findings and Investigations:**

Ultrasonography and contrast-enhanced MRI revealed intramuscular hemangioma involving the right splenius capitis muscle with no extension to adjacent muscles and minimal extension to the subcutaneous tissue.

**Interventions and Outcome:**

Excision of the lesion along with splenius capitis was performed with stable postoperative hemodynamics.

**Conclusion:**

Since intramuscular hemangiomas pose a challenge in preoperative diagnosis, it requires the sensible use of imaging modalities. Although several treatment modalities have surfaced, intramuscular hemangiomas require definitive operative management to reduce their recurrence.

## Introduction

Highlights

- Intramuscular hemangiomas are rare benign tumors that account for 1% of all hemangiomas and are even more rarer and uncommon in the head and neck region.
- Intramuscular hemangiomas have a variable presentation, and accurate preoperative diagnosis has been reported in less than 8% of cases because bruits, thrills, and pulsations are frequently masked due to muscular fibrosis concealing the vascular nature.
- The definitive diagnosis can be made through the histological study of surgical or biopsy specimens. However, MRI is the gold standard for preoperative diagnosis as it allows better delineation of the tumor.
- Many treatment modalities have been used for the management of such cases; however, definitive surgical management is required. Such tumors have a high recurrence rate, and the local recurrence rate can be maintained through wide excision, including the cuff of surrounding muscle.

Hemangiomas, although being the most common benign tumor, arising within skeletal muscle account for less than 1% of all hemangiomas and occur in large musculature of the trunk and extremities^1^,^2^. Intramuscular hemangiomas are uncommon in the head and neck region (14–21% of intramuscular hemangiomas) and mostly involve the masseter muscle^1^–^3^. Intramuscular hemangiomas are nonencapsulated benign neoplasms involving skeletal muscle and deep peripheral soft tissue that are composed of vascular channels along with adipose tissue, fibrous, and myxoid tissue^2^,^3^. Intramuscular hemangiomas occur mostly in childhood with 85% noted by the age of 1 year^4^,^5^ with no sex preference^6^,^7^. Only 8% of the cases have been reported with the exact preoperative diagnosis because bruits, thrills, and pulsations are frequently masked due to muscular fibrosis^1^.

Herein we report a rare case of intramuscular hemangioma of splenius capitis that was managed successfully with the excision of the lesion along with the muscle involved.

## Method

We reported this case following the updated consensus-based Surgical CAse REport (SCARE) guidelines^8^.

## Case presentation

A 20-year-old male army personnel belonging to the Kirati ethnicity with no known comorbidities presented to the neurosurgery outpatient department of our hospital (a tertiary care facility) with the chief complaint of swelling over the nape of the neck on the right side. According to the patient, the swelling first presented at 20 days of his life, which was the size of a pea that gradually increased to a size of a tennis ball spontaneously. There was no history of trauma. His past history, family history, drug history, and allergic history are nonremarkable. He is a nonsmoker, does not consume alcohol, and has normal sleep and bowel habit. On clinical examination, the solitary swelling was 4×4 cm on measurement, soft on consistency with regular margin, fluctuant, with no skin changes over the swelling, nontender, no restriction in range of motion of the neck, and no pulsation felt. His vital parameters were normal, with no significant findings in systemic examinations.

**Figure 1:**
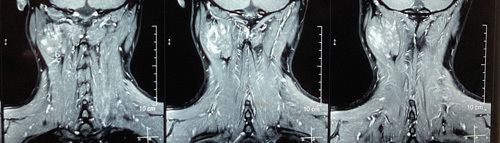
MRI of the cervical spine; T1-weighted scan (coronal section) showing intramuscular hemangioma of splenius capitis.

**Figure 2:**
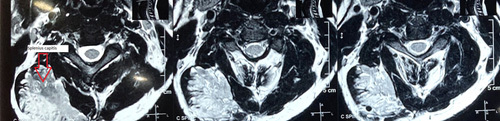
MRI of the cervical spine; T2-weighted scan (axial section) showing intramuscular hemangioma of splenius capitis (red arrow) with no extension to the adjacent muscle.

Ultrasonography was ordered, which showed an ill-defined anechoic cystic lesion with thick echogenic internal septation and strands in the subcutaneous plane. A contrast-enhanced MRI revealed fat saturated heterogeneously high signal intensity intramuscular lesion suggesting intramuscular hemangioma involving the right splenius capitis muscle with no extension to adjacent muscles and minimal extension to the subcutaneous tissue (Figs. 1, 2).

**Figure 3:**
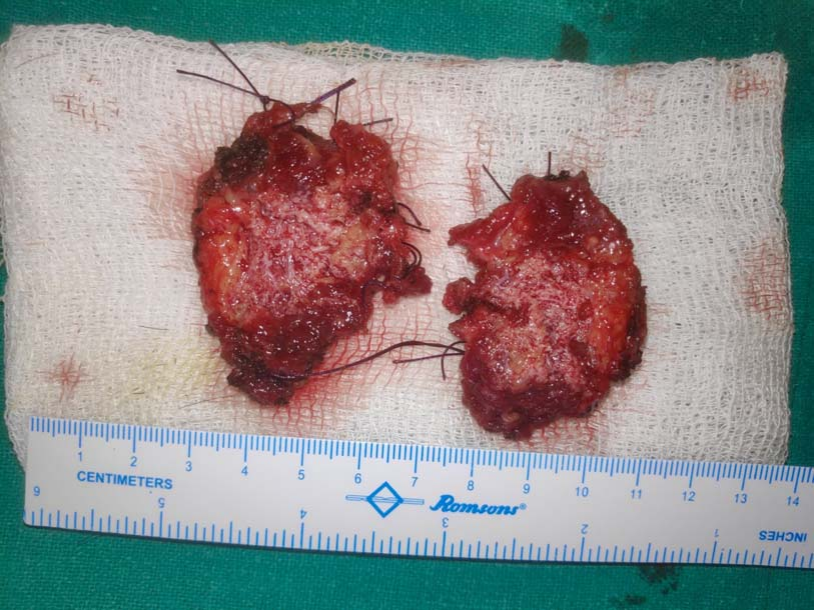
Gross specimen of intramuscular hemangioma postoperatively.

All of his laboratory parameters were within the normal range. He was then scheduled for surgery. The patient was informed about the risks and benefits of the procedure, along with an explanation of other alternative procedures. He wanted the lesion to be surgically removed. Thus, he underwent excision of the lesion along with the excision of splenius capitis with minimal blood loss. The specimen (Fig. 3) was sent for histopathology examination, which showed prominent arteriovenous and lymphatic components associated with adipose tissue dissecting through skeletal muscle fibers with no malignant features (features consistent with an intramuscular hemangioma with a predominant arteriovenous component). He was shifted to the postoperative ward with stable hemodynamic parameters. On his second postoperative day, he was on a liquid diet, and he had decreased mobility of the neck due to the surgery. His diet was changed to solids on third postoperative day. He was discharged from the hospital on his third postoperative day because he was hemodynamically stable and asymptomatic. During his scheduled follow-up after a month of discharge, he was asymptomatic with a normal range of motion on his neck and no signs of any new swelling or recurrence.

## Discussion

Intramuscular hemangiomas are noticed around adulthood once visible symptoms occur like swelling, pain, or cosmetic asymmetry, although they occur predominantly in childhood^4^. Hemangiomas can mostly be nonvascular and spongy (capillary type), composed of large vessels lined by flattened endothelial cells (cavernous type) or mixed^3^,^9^,^10^. However hemangiomas are classified into many categories according to the Modified International Society for the Study of Vascular Anomalies classification^11^. Intramuscular hemangiomas have controversial origins ranging from congenital and traumatic to hormonal theories^2^. Intramuscular hemangiomas are postulated to arise from congenital cutaneous lipomas with hormonal influences. Fat necrosis with hemorrhagic conversion due to minor trauma and hormonal influence result in the vascular component of the tumor^3^.

Since intramuscular hemangiomas are rare, they possess considerable therapeutic challenges regarding their locations^9^. A palpable mass is an initial presentation and maybe the only presentation in most cases with the presence of mobility in a plane perpendicular to the line of muscle^1^. Cavernous-type mass can be enlarged by an increase in venous pressure through the Valsalva maneuver, recumbence, bending forward, or jugular compression^12^. This variability of presentations makes physical examination an unreliable tool for diagnosis. Inaccurate preoperative diagnosis leads to inappropriate treatment planning that causes unnecessary complications.

The definitive diagnosis can be made through a histological examination of the specimen^9^. Soft tissue density encountered in the region of skeletal muscle should prompt consideration of the diagnosis of intramuscular hemangioma^9^. Surgical diagnoses like fine-needle aspirations and incisional biopsies include a risk of unnecessary bleeding; thus, MRI is the gold standard preoperative diagnostic tool that demonstrates a heterogeneously increased signal within the lesion. Also, MRI allows better delineation of tumor components, degree of the local extension, and differentiation in comparison to computed tomography scan^3^. Arteriography is practical only if the tumor is large and there is suspicion of large vascular connections^1^,^13^.

Spontaneous regression of the tumor does not occur^14^, and management thus should be individualized based on tumor location, depth of invasion, accessibility, age, and cosmesis^12^,^13^. Wide excision, including the cuff of surrounding muscle, is generally the preferred treatment of choice to prevent local recurrence^9^,^14^. In all, 92% local recurrence-free survival can be achieved through wide excision, which drops to 65% with intralesional excision but with positive microscopic margins^15^. Local recurrence-free survival in 5 years drops to 33% where complete excision of all gross tumors cannot be excised^15^. Recent advances in treatment are laser-based therapies, cryotherapy, sclerotherapy, and radiation to shrink the larger lesion to make the resection smoother along with reducing functional and cosmetic defects^3^. Also, systemic steroids have been used for the treatment of a few cases of such lesions to decrease the tumor bulk^1^. However, definitive surgical management is required.

Intramuscular hemangiomas, although benign and have not been reported to metastasize, have a higher recurrence rate of about 9–28% of the cases due to the microscopically infiltrative pattern of diffusion into surrounding muscular planes of least resistance, thus requiring long-term clinical and radiological follow-ups^4^,^16^. Due to the lower recurrence rate of wide excision and the fact that it is also more effective in removing the lesion as a whole than the other alternative, our patient chose the surgical route.

## Conclusion

Intramuscular hemangiomas usually appear as an enlarging mass without specific clinical features. While a superficial mucosal lesion can be diagnosed on clinical grounds, deep lesions require imaging modalities. This case of intramuscular hemangioma is reported to show the presentation of such a lesion, which might help surgeons be aware of the possibility of such a lesion. Hence, clinicians should have a high degree of clinical suspicion and make judicious use of imaging modalities for the diagnosis of the lesion.

Such a lesion requires definitive operative management and regular follow-ups.

## Ethical approval

None.

## Patient consent

Written informed consent was obtained from the patient for the publication of this case report and accompanying images. A copy is available for review by the Editor-in-Chief of this journal on request.

## Source of funding

None.

## Conflicts of interest disclosure

There are no conflicts of interest.

## Data availability statement

Not applicable.

## Provenance and peer review

Not commissioned, externally peer-reviewed.

## Declaration of interest statement

None.
